# Supplementary material for: Heat adaptation of phage T7 under an extended genetic code
Source: Virus Evol. 2021 Dec 1;7(2):veab100. doi: 10.1093/ve/veab100 (PMC8923235; doi:10.1093/ve/veab100)
Supplement: veab100_Supp [file veab100_supp.zip › Sequencing_Analysis.zip!/Sequencing_Analysis/A1/evidence/JC_SIDE_1_64.html]

BRESEQ :: Evidence


| New junction evidence | | | | | | | | | | | |
| --- | --- | --- | --- | --- | --- | --- | --- | --- | --- | --- | --- |
|  | | seq id | position | reads (cov) | reads (cov) | score | skew | freq | annotation | gene | product |
| \* | ? | NC\_001604.1 | = 27753 | 2681 (0.920) | 176 (0.060) +CGCTATATCTGG | 45/544 | 24.3 | 6.0% | intergenic (–/–) | *–/–* | –/– |
|  | ? | NC\_001604.1 | 27754 = | 2915 (0.940) |  |  |  |  | intergenic (–/–) | *–/–* | –/– |
| Rejected: Coverage evenness skew score above cutoff. | | | | | | | | | | | |
| Rejected: Frequency below/above cutoff threshold. | | | | | | | | | | | |

Only 100 of 3084 total aligned reads displayed.  

`CTAGCTATCGGTGGTAACTGCGGGGACCAGTGCTGGTTCGTTACGAGCGACCAAGTGTGGCGACTTAGTGGAAAGGCTAAGCGAAAGTTCCGTAAGTTAATCATGGAGTATCGCGATAAGATGCTTGAGAAGTATGATACTCTTTGGAATTACGTATGGGTAGGCAATACGTCCCACATTCGTTTCCTCAAGACTATCGGTGCGGTATTCCATGAAGAGTACACACGAGATGGTCAATTTCAGTTATTTACAATCACGAAAGGAGGATAACCATATGTGTTGGGCAGCCGCAA‑‑‑TACCTATCGCTATATCTGGCGCTCAGGCTATCAGTGGTCAGAACGCTCAGGCCAAAATGATTGCCGCTCAGACCGCTGCTGGTCGTCGTCAAGCTATGGAAATCATGAGGCAGACGAACATCCAGAATGCTGACCTATCGTTGCAAGCTCGAAGTAAACTTGAGGAAGCGTCCGCCGAGTTGACCTCACAGAACATGCAGAAGGTCCAAGCTATTGGGTCTATCCGAGCGGCTATCGGAGAGAGTATGCTTGAAGGTTCCTCAATGGACCGCATTAAGCGAGTCACAGAAGGACAGTTCATTCGGGA  >  NC_001604.1/27454‑28053`  

                                                                                                                                                                                                                                                                                                              `|`                                                                                                                                                                                                                                                                                                              
              `tAACTGCGGGGACCAGTGCTGGTTCGTTACGAGCGACCAAGTGTGGCGACTTAGTGGAAAGGCTAAGCGAAAGTTCCGTAAGTTAATCATGGAGTATCGCGATAAGATGCTTGAGAAGTATGATACTCTTTGGAATTACGTATGGGTAGGCAATACGTCCCACATTCGTTTCCTCAAGACTATCGGTGCTGTATTCCATGAAAAGTACACACGAGATGGTCAATTTCAGTTATTTACAATCACAAAAAGAGGATAACCATATGTGTTGGGAAGCCTCAA‑‑‑TACCTAACGCTATAACttgagc                                                                                                                                                                                                                                                                                               >  1:129615/1‑296 (MQ=255)`  
                `aCTGCGGGGACCAGTGCGGGTTCGTTACGAGCGACCAAGTGTGGCGACTTAGTGGAAAGGCTAAGCGAAAGTTCCGTAAGTTAATCATGGAGTATCGCGATAAGATGCTTGAGAAGTATGATACTCTTTGGAATTACGTATGGGTAGGCAATACGTCCCACATTCGTTTCCTCAAGACTATCGGTGCGGTATTCCATGAAGAGTACACACGAGATGGTCAATTTCAGTTATTTACAATCACGAAAGGAGGATAACCATATGTGTTGGGCAGCCGCAA‑‑‑TACCTATCGCTATATCTGGCGCTc                                                                                                                                                                                                                                                                                             <  1:351518/301‑1 (MQ=255)`  
                 `cTGCGGGGACCAGTGCTGGTTCGTTACGAGCGACCAAGTGTGGCGACTTAGTGGAAAGGCTAAGCGAAAGTTCCGTAAGTTAATCATGGAGTATCGCGATAAGATGCTTGAGAAGTATGATACTCTTTGGAATTACGTATGGGTAGGCAATACGTCCCACATTCGTTTCCTCAAGACTATCGGTGCGGTATTCCATGAAGAGTACACACGAGATGGTCAATTTCAGTTATTTACAATCACGAAAGGAGGATAACCATATGTGTTGGGCAGCCGCAA‑‑‑TACCTATCGCTATATCTGGCGCtaa                                                                                                                                                                                                                                                                                            >  1:254518/1‑299 (MQ=255)`  
                        `gACCAGTGCTGGTTCGTTACGAGCGACCAAGTGTGGCTACTTAGTGGAAAGGCCAATCGAAAGTTCCGTAAGTTAATCATGGAGTATCGCGATAAGATGCTTGAGAAGTATGATACTCTTTGGAATTACGTATGGGTTGGCAATACGTCCCACATTCGTTTCCTCAAGACTATCGGTGCGGTATTCCATGAAGAGTACACACGAGATGGTCAATTTCAGTTATTTACAATCACGAAAGGAGGATAACCATATGTGTTGGGCAGCCGCAA‑‑‑TACCTATCGCTATATCTGGCGCTCAGGCTATc                                                                                                                                                                                                                                                                                     <  1:143620/301‑1 (MQ=255)`  
                          `ccAGTGCTGGTTCGTTACGAGCGACCAAGTGTGGCTACTTAGTGGAAAGGCTAAGCGAAAGTTCCGTAAGTTAATCATGGAGTATCGCGATAAGATGCTTGAGAAGTATGATGCTCTTTGGAATTACGTATGGGTAGGCAATACGTCCCACATTCGTTTCCTCAAGACTATCGGTGCGGTATTCCATGAAGAGTACACACGAGATGGTCAATTTCAGTTATTTACAATCACGAAAGGAGGATAACCATATGTGTTGGGCAGCCGCAA‑‑‑TACCTATCGCTATATCTGGCGCTCAGGCTATCAg                                                                                                                                                                                                                                                                                   <  1:475901/301‑1 (MQ=255)`  
                          `ccAGTGCTGGTTCGTTACGAGCGACCAAGTGTGGCGACTTAGTGGAAAGGCTAAGCGAAAGTTCCGTAAGTTAATCATGGAGTATCGCGATAAGATGCTTGAGAAGTATGATACTCTTTGGAATTACGTATGGGTAGGCAATACGTCCCACATTCGTTTCCTCAAGACTATCGGTGCGGTATTCCATGAAGAGTACACACGAGATGGTCAATTTCAGTTATTTACAATCACGAAAGGAGGATAACCATATGTGTTGGGCAGCCGCAA‑‑‑TACCTATCGCTATATCTGGCGCTCAGGCTATCAg                                                                                                                                                                                                                                                                                   <  1:406856/301‑1 (MQ=255)`  
                             `gTGCTGGTTCGTTACGAGCGACCAAGTGTGGCGACTTAGTGGAAAGGCTAAGCGAAAGTTCCGTAAGTTAATCATGGAGTATCGCGATAAGATGCTTGAGAAGTATGATACTCTTTGGAATTACGTATGGGTAGGCAATACGTCCCACATTCGTTTCCTCAAGACTATCGGTGCGGTATTCCATGAAGAGTACACACGAGATGGTCACTTTCAGTTATTTACACTCACGAAAGTAGGATAACCATATGTGTTGGGCAGCCGCAA‑‑‑TACCTCTCGCTATATCTGGCGCACAGGCAATCCGTgt                                                                                                                                                                                                                                                                                >  1:52744/1‑300 (MQ=255)`  
                                  `ggTTCGTTACGGGCGACAAAGTGTGGCGACTTAGTGGAAAGGCTAGGCGAAAGTTCCGTAAGTTAATCATGGAGTATCGCGATAAGATGCTTGAGAAGTATGATACTCTTTGGAATTACGTATGGGTAGGCAATACGTCCCACATTCGTTTCCTCAAGACTATCGGTGCGGTATTCCATGAAGAGTACACACGAGATGGTCAATTTCAGTTATTTACAATCACGAAAGGAGGATAACCATATGTGTTGGGCAGCCGCAA‑‑‑TACCTATCGCTATATCTGGCGCTCAGGCTATCAGTGATCAGa                                                                                                                                                                                                                                                                           <  1:373002/301‑1 (MQ=255)`  
                                             `aGCGACCAAGTGTGGCGACTTAGTGGAAAGGCTAAGCGAAAGTTCCGTAAGTTAATCATGGAGTATCGCGATAAGATGCTTGAGAAGTATGATACTCTTTGGAATTACGTATGGGTAGGCAATACGTCCCACATTCGTTTCCTCAAGACTATCGGTGCGGTATTCCATGAAGAGTACACACGAGATGGTCAATTTCAGTTATTTACAATCACGAAAGGAGGATAACCATATGTGTTAGGCAGCCGCAA‑‑‑TACCTATCGCTATATCTGGCGCTCCGGCTATCAGTGATCAGAACGCTCactgt                                                                                                                                                                                                                                                                >  1:383036/1‑297 (MQ=255)`  
                                                 `aCCAAGTGTGGCGACTTAGTGGAAAGGCTAAGCGAAAGTTCCGTAAGTTAATCATGGAGTATCGCGATAAGATGCTTTAGAAGTATGATACTCTTTGGACTTACGTATGGGTAGGCAATACGTCCCACATTCGTTTCCTCAAGACTATCGGTGCGGTATTCCATGCAGAGTACACACGAGATGGTCAATTTCAGTTATTTACAATCACGAAAGGAGGATAACCATATGTGTTGGGCAGCCGCAA‑‑‑TACCTATCGCTATATCTGGCGCTCAGGCTATCAGTgctgtctcttatacacatctcc                                                                                                                                                                                                                                                            >  1:210796/1‑280 (MQ=255)`  
                                                                  `aGTGGAAAGGCTAAGCGAAAGTTCCGTAAGTTAATCATGGAGTATCGCGATAAGATGCTTGAGAAGTATGATACTCTTTGGAATTACGTATGGGTAGGCAATACGTCCCACATTCATTTCCTCAAGACTATCGGTGCGGTATTCCATGAAGAGTACACACGAGATGGTCAATTTCAGTTATTTACAATCACGAAAGGAGGATAACCATATGTGTTGGGCAGCCGCAA‑‑‑TACCTATCGCTATATCTGGCGCTCAGGCTATCAGTGATCAGAACGCTCAGGCCAAAATGATTGCCGCTCAGAcc                                                                                                                                                                                                                                           <  1:454992/301‑1 (MQ=255)`  
                                                                                      `gTTCCGTAAGTTAATCATGGAGTATCGCGATAAGATGCTTGAGAAGTATGATACTCTTTGGAATTACGTATGGGTAGGCAATACGTCCTACATTCGTTTCCTCAAGACTATCGGTGCGGTATTCCATGAAGAGTACACACGAGATGGTCAATTTCAGTTATTTACAATCACGAAAGGAGGATAACCATATGTGTTGGGCAGCCGCAA‑‑‑TACCTATCGCTATATCTGGCGCTCAGGCTATCAGTGATAAGAACGCTCAGGCCAAAATGATTGCCGCTCAGACCGCTGCTGGTCGTCGTCAAGc                                                                                                                                                                                                                       >  1:411690/1‑301 (MQ=255)`  
                                                                                      `gTTCCGTAAGTTAATCATGGAGTATCGCGATAAGAGGCTTGAGAAGTATGATACTCTTTGGAATTACGTATGGGTAGGCAATACGTCCCACATTCGTTTTATCAAGACTATCGGTGCGGTATTCCATGAAGAGTACACACGAGATGGTCAATTTCAGTTATTTACAATCACGAAAGGAGGTTAACCATATGTGTTGGGCAGCCGCAA‑‑‑TACCTATCGCTATATCTGGCGCTCAGGCTATCAGTGATCAGAACGCTCAGGCCAAAATGATTGCCGCTCAGACCGCTGCTGGTCGTCGTCAAGc                                                                                                                                                                                                                       <  1:338533/301‑1 (MQ=255)`  
                                                                                       `ttCCGTAAGTTAATCATGGAGTATCGCGATAAGATGCTTGATAAGTATGATACTCTTTGGAATTACTTATGGGTAGGCAATACGTCCCACATTCGTTTCCTCAAGACTATCGGTGCGGTATTCCATGAAGAGTACACACGAGATTGTCAATTTCAGTTATTTACAATCACGAAAGGAGGATAACCATATGTGTTGGGCAGCCGCAA‑‑‑TACCTATCGCTATATCTGGCGCTCAGGCTATCAGTGATCAGAACGCTCAGTCCAAAATGATTGCCGCTCAGACCGCTGCTGGTCGTCGTCAAGCt                                                                                                                                                                                                                      <  1:100077/301‑1 (MQ=255)`  
                                                                                                   `aTCATGGAGTATCGTGATAAGATGCTTTAGAAGGATGATACTCTTTGGAAATACGTATGGGTAGGCAATACGTCCCACATTTGTTTCCTAAAGACGATCGGTTCGGTATTCCATGAAGAGTACACACGAGATGGTCAATTTAAGTTATTTACAATCACGAAAGGAGGATAACCATATGTGTTGGGCAGCCTCAA‑‑‑TACCTATCGCTATATCTGGCGCTCAGGCTATCAGTGATCAGAACGCTCAGGCCAAAATGATTGCCGCTCAGACCGCTGCTGGTCGTCGTCAAGCTATGGAAATCATg                                                                                                                                                                                                          <  1:432473/301‑1 (MQ=255)`  
                                                                                                             `aTCGCGATAAGATGCTTGAGAAGTATGATACTCTTTGGAATTACGTATGGGTAGGCAATACGTCCCACATTCGTTTCCTCAAGACTATCGGTGCGATATTCCATGAAGAGTACACACGAGATGGTCAATTTCAGTTATTTACAATCACGAAAGGAGGATAACCATATGTGTTGGGCAGCCGCAA‑‑‑TACCTATCGCTATATCTGGCGCTTAGGCTATCAGTGATCAGAACGCTCAGTCCAAAATCATTGCCGCTCACACCGCTGCTGGGCGTCGTCCAGCTATGGAAATCTTGCGCAAGACgg                                                                                                                                                                                                >  1:361722/1‑300 (MQ=255)`  
                                                                                                                   `gtataagagacaggaATTATGCTACTCTTTGGAATTACGTATGGGTAGGCAATACGTCCCACATTCGTTTCCTCAAGACTATCGGTTCGGTATTCCATGAAGAGTACACACGAGATGGTCAATTTCAGTTATTTACAATAACGAAAGGAGGATAACCATATGTGTTGGGCAGCCGCAA‑‑‑TACCTATCGCTATATCTGGCGCTCAGGATATCAGTGATCAGAACGCTCAGGCCAAAATGATTGCCGCTCAGACCGCTGCTGGTCGTCGTCAAGCTATGGAAATCATGAGGCAGACGAACATcc                                                                                                                                                                                          <  1:320962/288‑1 (MQ=255)`  
                                                                                                                   `aTAAGATGCTTGAGAAGTATGATACTCTTTGGAATTACGTATGGGTAGGCAATACGTCCCACATTCGTTTCCTCAAGACTATCGGTGCGGTATTCCTTGAAGAGTACACACGAGATGGTCAATTTCAGTTATTTACAATCACGAAAGGAGGATAACCATATGTGTTGGGCTGCCGCAA‑‑‑TACCTATCGCTATATCTGGCGCTCAGGCTATCAGTGATCAGAACGCTCAAGCCAAAATGATTGCCGCTCAGACCGCTGCTGGTCGTCGTCAAGCTATGGAAATCATGAGGCAGACGACCATcc                                                                                                                                                                                          >  1:215512/1‑301 (MQ=255)`  
                                                                                                                        `aTGCTTGAGAAGTATGATACTCTTTGGAATTACGTATGGGTAGGCAATACGTCCCACATTCGTTTCCTCAAGACTATCGGTGCGGTATTCCATGAAGAGTACACACGAGATGGTCAATTTCAGTTATTTACAATCACGAAAGGAGGATAACCATATGTGTTGGGCAGCCGCAA‑‑‑TACCTATCGCTATATCTGGCTCTCAGGCTATCAGTGATCAGAACGCTCAGGCCAAAATGATTGCCGCTCAGACCGCTGCTGGTCGTCGTCAAGCTATGGAAATCATGAGGCAGACGAACATCCAGaag                                                                                                                                                                                     >  1:307961/1‑300 (MQ=255)`  
                                                                                                                              `gagaAGTATGATAATCTTTGGAATTACGTATGGGTAGGCAATACGTCCCACATTCGTTTCCTCAAGACTATCGGTGCGGTATTCCATGAAGAGTACAAACGAGATGGTCAATTTCAGTTATTTACAATCACGAAAGGAGGATAACCATATGTGTTGGGCAGCCGCAA‑‑‑TACCTATCGCTATATCTGGCGCTCAGGCTATCAGTGATCAGAACGCTCAGGCCAAAATGATTGCCGCTCAGACCGCTGCTGGTCGTCGTCAAGCTATGGAAATCATGAGGCAGACGAACATCCAGAATGCTGAc                                                                                                                                                                               <  1:241180/301‑1 (MQ=255)`  
                                                                                                                                 `acgTATGATACTCTTTGGAATTCCTTCTTTTTCTCCAATCCTTCCCACATTCGTTTCCTCAAGACTATCGGTGCGGTATTCCATGAAGAGTACACACGAGATGGACAATTTCAGTTATTTACAATCACGAAAGGAGGATAATCATATGTGTTGGGCAGCCGCAA‑‑‑TACCTATCGCAATATCTGGCGCTCAGGTTATCAGTGATCAGAACGCTCAGGCCAGAATGATTGCCGCTCAGACCGCTGCTGGTCGTCGTCAAGCTATGGAAATAATGAGGAAGACGACCACCCAgctgcctcttata                                                                                                                                                                            >  1:447490/3‑289 (MQ=255)`  
                                                                                                                                  `aGTATGATACTCTTTGGAATTACGTATGGGTAGGCAATACGTCCCACATTCGTTTCCTCAAGACTATCGGTGCGGTATTCCATGAAGAGTACACACGAGATGGTCAATTTCAGTTATTTACAATCACGAAAGGAGGATAACCATATGTGTTGGGCAGCCGCAA‑‑‑TACCTATCGCTATATCTGGCGCTCAGGCTATCAGTGATCAGAACGCTCAGGCCAAAATGATTGCCGCTCAGACCGCTGCTGGTCGTCGTCAAGCTATGGAAATCATGAGGCAGACGAACATCCAGAATGCTGACCTAt                                                                                                                                                                           <  1:458787/301‑1 (MQ=255)`  
                                                                                                                                   `gTATGATACTCTTTGGAATTACGTATGGGTAGGCAATACGTCCCACATTCGTTTACTCAAGACTATCGGTGCGGTATTCCATGAAGAGTACACACGAGATGGTCAATTTCAGTTATTTACAATCACGAAAGGAGGATAACCATATGTGTTGGGCAGCCGCAC‑‑‑TACCTATCGCTATATCTGGAGCTCAGGCTCTCAGTGATCAGAACGCTCAGGCCAAAATGATTGCCGCTCAGAACGCTGCTGGTCGTCGACAAGCTATAGAAATCATTCGGCCGCCGGACACACAGAAAGCTGcccctgt                                                                                                                                                                          >  1:148789/1‑297 (MQ=255)`  
                                                                                                                                   `gTATGATACTCTTTGGAATTACGTATGGGTAGGAAATACGTCCCACATTCGTTTCCTCAAGACTATCGGTGCGGTATTCCATGAAGAGTACACACGAGATGGTCAATTTCAGTTATTTACAATCACGAAAGGAGGATAACCATATGTGTTGGGCAGCCGCAA‑‑‑TACCTATCGCTATATCTGGCGCTCAGGCTATCAGTGATCAGAACGCTCAGGCCAAAATGATTGCCGCTCAGACAGCTGCTGGTCGTCGTCAAGCTATGGAAATCATGAGGCAGACGAACATCCAGAATGCTGACCTaac                                                                                                                                                                          >  1:119630/1‑299 (MQ=255)`  
                                                                                                                                    `tATGATACTCTTTGGAATTACGTATGGGTAGGCAATACGTCCCACATTCGTTTCCTCAAGACGATCGGTGCGGTATTCCATGAAGAGTACACACGAGATGGTCAATTTCAGTTATTTACAATCACGAAAGGAGGATAACCATATGTGTTGGGCAGCCGCAA‑‑‑TACCTATCGCTATATCTGGCGCTCAGGCTATCAGTGATCAGAACGCTCAGGCCAAAATGATTGCCGCTCAGACCGCTGCTGGTCGTCGTCAAGCTATGGAAATCATGAGGCAGACGAACATCCAGAATGCTGACCTATCg                                                                                                                                                                         <  1:471236/301‑1 (MQ=255)`  
                                                                                                                                     `aTGATACTCTTTGGAATTACGTATGGGTAGGCAATACGTCCCACATTCGTTTCCTCAATACTATCGGTGCGTTATTCCATGCAGCGTACACACGAGATGGTCAATTTCAGTTATTTACAATCACGAAAGGAGGATAACCATCTGTGTTGGGCAGCCTCAA‑‑‑TACCTATCGCTATCTCTGGCGCTCAGGCTATCAGTGATCAGAACGCTCAGGCCAAAATGATTGCCGCTCAGACCGCTGCTGGTCGTCGTCACGCTATGGAAATCATGAGGCAGACGAACATCCAGAATGCTTACCTATCGt                                                                                                                                                                        >  1:56958/1‑301 (MQ=255)`  
                                                                                                                                         `tACTCTTTGGAATTACGTATTGGTAGGCAATACGTCCCACATTCGTTTCCTCAAGACTATCGGTGCGGTATTACATGAAGAGTACACACGAGATGGTCAATTTCAGTTATTTACAATCACGAAATGAGGATAACCATATGTGTTGGGCAGCCGCAA‑‑‑TACCTATCGCTATATCTGGCGCTCAGGCTATCAGTGATCAGAACGCGCAGGCCAAAATGATTGCCGCTCAGACCGCTGCTGGTCGTCGTCAAGCTATGGAAATCATGAGGCAGACGAACATCCAGAATGCTGACCTATCGTTGCa                                                                                                                                                                    <  1:316360/301‑1 (MQ=255)`  
                                                                                                                                                       `aCGTATTGGTAGGCAATACGTCCCACATTCGTTTCCTCAAGACTATCGGTGCGGTATTCCATGAAGAGTACACACGAGATGGTCAATTTCAGTTATTTACAATCACGAAAGGAGGATAACCATATGTGTTGGGCAGTCGCAA‑‑‑TACCTATCGCTATATCTGGCGCTCAGGCTATCAGTGATCAGAACGCTCAGGCCAAAATGATTGCCGCTCAGACCGCTGCTGGTCGTCGTCAAGCTATGGAAATCATGAGTCAGACGAACATCCAGAATGCTGACCTATCGTTGCAAGCTCGAAGTAAAc                                                                                                                                                      >  1:197240/1‑301 (MQ=255)`  
                                                                                                                                                       `aCGTATGGGTAGGCAATACGTCCCACATTCGTTTCCTCAAGACTATCGGTGCGGTATTCCATGAAGAGTACACACGAGATGGTCAATTTCAGTTATTTACAATCACGAAAGGAGGATAACCATATGTGTTGGGCAGCCGCAA‑‑‑TACCTATCGCTATATCTGGCGCTCAGGCTATCAGTGATCAGAACGCTCAGGCCAAAATGATTGCCGCTCAGACCGCTGCTGGTCGTCGTCAATCTATGGAAATCATGAGGCAGACGAACATCCAGAATGCTGACCTATCGTTGCAAGCTCGAAGTAAAc                                                                                                                                                      >  1:427558/1‑301 (MQ=255)`  
                                                                                                                                                            `tGGGTAGGCAATACGTCCCACATTCGTTTCCTCAAGACTATCGGTGCGGTATTCCATGAAGAGTACACACGAGATGGTCAATTTCAGTTATTTACAATCACGAAAGGAGGATAACCATATGTGTTGGGCAGCCGCAA‑‑‑TACCTATCGCTATATCTGGCGCTCAGGCTATCAGTGATCAGAACGCTCAGGCCAAAATGATTGCCGCTCAGACCGCTGCTGGTCGTCGTCAAGCTATGGAAATCATGAGGCAGACGAACATCCAGAATGCTGACCTAACGTTGCAAGCTCGAAGTAAGCTTGAg                                                                                                                                                 >  1:333568/1‑301 (MQ=255)`  
                                                                                                                                                             `gggTAGGCAATACGTCCCACATTCGTTTCCTCAAGACTATCGGTGCGGTATTCCATGAAGAGTACACACGAGATGGTCAATTTCAGTTATTTACAATCACGAAAGGAGGATAACCATATGTGTTGGGCAGCCGCAA‑‑‑TACCTATCGCTATATCTGGCGCTCAGGCTATCAGTGATCAGAACGCTCAGGCCAAAATGATTGCCGCTCAGACCGCTGCTGGTCGTCGTCAAGCTATGGAAATCATGAGGCAGACGAACATCAAGAATGCTGACCTATCGTTGCAAGCTCGAAGTAAACTTGAgg                                                                                                                                                >  1:5044/1‑301 (MQ=255)`  
                                                                                                                                                                 `agacAATACGTCCCACATTCGTTTCCTCAAGACTATCGGTGCGGTATTCCATGAAGAGTACACACGAGATGGTCAATTTCAGTTATTTACAATCACGAAAGGAGGATAACCATATGTGTTGGGCAGCCGCAA‑‑‑TACCTATCGCTATATCTGGCGCTCAGGCTATCAGTGATCAGAACGCTCAGGCCAAAATGATTGCCGCTCAGACCGCTGCTGGTCGTCGTCAAGCTATGGAAATCATGAGGCCGACGAACATCCAGAATGCTGACCTATCGTTGCAAGCTCGAAGTGAACtctgcctctt                                                                                                                                            >  1:176755/4‑292 (MQ=255)`  
                                                                                                                                                                  `ggCAATACGTCCCACATTCGTTTCCTCAAGACTATCGGTGCGGTATTCCATGAAGAGTACACACGAGATGGTAAATTTCAGTTATTTACAATCACGAAAGGAGGATAACCATATGTGTTGGGCAGCCGCAA‑‑‑TACCTATCGCTATATCTGGCGCTCAGGCTATCAGTGATCAGAACGCTCAGGCCAAAATGATTGCCGCCCAGACCGCTGCTGGTCGTCGTCAAGCTATGGAAATCATGAGGCAGACGAACATCCAGAATGCTGACCTATCGTTGCAAGCTCGAAGTAAACTTGAGGAAGCg                                                                                                                                           >  1:451092/1‑301 (MQ=255)`  
                                                                                                                                                                            `cccATATTCGTTTCCTCAAGACTATCGGTGCAGTATTCCATGAAGAGTACACACGAGATGGTCAATTTCAGTTATTTACAATCACGAAAGGAGGATAACCATATGTGTTGGGCAGCCGCAA‑‑‑TACCTATCGCTATATCTGGCGCTCAGGCTATCAGTGATCAGAACGCTCAGGCCAAAATGATTGCCGCTCAGACCGCTGCTGGTCGTCGTCAAGCTATGGAAATCATGAGGCAGACGAACATCCAGAATGCTGACCTATCGTTGCAAGCTCGAAGTAAACTTGAGGAAGCGTCCGCCGAgg                                                                                                                                 >  1:274131/1‑300 (MQ=255)`  
                                                                                                                                                                             `ccACATTCGTTTCCTCAAGACTATCGGTGCGGTATTCCATGAAGAGTACACACGATATGGTCAATTTCAGTTATTTACAATCACGAAAGGAGGATAACCATCTGTGTTGGGCAGCCGCAA‑‑‑TACCTATCGCTATATCTGGCGCTCAGGCTATCAGTGATCAGAACGCTCAGGCCACAATGATTGCCGCTCAGACCGCTGCTGGTCGTCGTCAAGCTATGGAAATCATGAGTCAGACGAACATCAAGAATGCTGACCTATAGTTGCAATCGCGAAGACAACTTGAGGAAGAGTCAGCCGAGtt                                                                                                                                >  1:326583/1‑301 (MQ=255)`  
                                                                                                                                                                             `ccACATTCGTTTCCTCAAGACTATCGGTGCGGTATTCCATGAAGAGTACACACGAGATGGTCAATTTCAGTTATTTACAATCACGAAAGGAGGATAACCATATGTGTTGGGCAGCCGCAA‑‑‑TACCTATCGCTATATCTGGCGCTCAGGATATCAGTGATCAGAACGCTCAGGCCAAAATGATTGCCGCTCAGACCGCTGCTGGTCGTCGTCAAGCTATGGAAATCATGAGGCAGACGAACATCCAGAATGCTGACCTATCGTTGCAAGCTCGAAGTAAACTTGAGGAAGCGTCCGCCGAGtt                                                                                                                                >  1:442324/1‑301 (MQ=255)`  
                                                                                                                                                                                       `ttCCTTAAGACTATCGGTGCGGTATTCCATGAAGAGTACACACGAGATGGTCAATTTCAGTTATTTACAATCACGAAAGGAGGATAACCATATGTGTTGGGCAGCCGCAA‑‑‑TACCTATCGCTATATCTGGCGCTCAGGCTATCAGTGATCAGAACGCTCAGGCCAAAATGATTGCCGCACAGACCGCTGCTGGTCGTCGTCAAGCTATGGAAATCATGAGGCAGACGAACATCCAGAATGCTGACCTATCGTTGCAAGCTCGAAGTAAACTTGAGGAAGCGTCCGCCGAGTTGACCCCACAg                                                                                                                      <  1:268584/301‑1 (MQ=255)`  
                                                                                                                                                                                          `aagagacagaTCGGTGCGGTATTCCATGAAGAGTACACACGAGATGGTCAATTTCAGTTATTTACAATCACGAAAGGAGGATAACCATATGTGTTGGACAGCCGCAA‑‑‑TACCTATCGCTATATCTGGCGCGCAGGCTATCAGTGATCAGAACGCTCAGGCCAAAATGATTGCCGCTCAGACCGCTGCTGGTCGTCGTCAAGCTATGGAAATCATGAGGCAGACGAACATCCAGAATGCTGACCTATCGTTGCAAGCTCGAAGTAATCTTGAGGAAGCGTCCGCCGAGTTGACCCCACAGAAc                                                                                                                   <  1:390734/292‑1 (MQ=255)`  
                                                                                                                                                                                               `ttataagagacaggTATTCCATGAAGAGTACACACGAGATGGTCAATTTCAGTTATTTACAATCACGAAAGGAGGATAACCATATGTGTTGGGCAGCCGCAA‑‑‑TACCTATCGCTATATCTGGCGCTCAGGCTATCAGTGATCAGAACGCTCAGGCCAAAATGATTGCCGCTCAGACCGCTGCTGGTCGTCGTCAAGCTATGGAAATCATGAGGCAGACGAACATCCAGAATGCTGACCTATCGTTGCAAGCTCGAAGTAAACTTGAGGAAGCGTCCGCCGAGTTGACCCCACAGAACATGCa                                                                                                              <  1:437690/289‑1 (MQ=255)`  
                                                                                                                                                                                                `aCTATCGGTGCGGTATTCCATGAAGAGTACACACGAGATGGTCAATTTCAGTTATTTACAATCACGAAAGGAGGATAACCATATGTGTTGGGCAGCCGCAA‑‑‑TACCTATCGCTATATCTGGCGCTCAGGCTATCAGTGATCAGAACGCTCAGGCCAAAATGATTGCCGCTCAGACCGCTGCTTGTCGTCGTCAAGCTATGGAAATCATGAGGCAGACGAACATCCAGAATGCTGACCTATCGTTGCAAGCTCGAAGTAAACTTGAGGAAGCGTCCGCCGAGTTGACCCCACAGAACATGCAg                                                                                                             <  1:377540/301‑1 (MQ=255)`  
                                                                                                                                                                                                   `aTCGGTGCGGTATTCCATGAAGAGTACACACGAGATGGTCAATTTCAGTTATTTACAATCACGAAAGGAGGATAACCATATGTGTTGGGCAGCCGCAA‑‑‑TACCTATCGCTATATCTGGCGCTCAGGCTATCAGTGATCAGAACGCTCAGGCCAAAATGATTGCCGCTCAGACCGCTGCTGGTCGTCGTCAAGCTATGGAAATCATGAGGCAGACGAACATCCAGAATGCTGACCTATCGTTGCAAGCTCGAAGTAAACTTGAGGAAGCGTCCGCCGAGTTGACCCCACAGAACATGCAGAAg                                                                                                          >  1:187556/1‑301 (MQ=255)`  
                                                                                                                                                                                                         `gCGGTATTCCATGAAGAGTACACACGAGATGGTCAATTTCAGTTATTTACAATCACGAAAGGAGGATAACCATATGTGTTGGGCAGCCGCAA‑‑‑TACCTATCGCTATATCTGGCGCTCAGGCTATCAGTGATCAGAACGCTCAGGCCAAAATGATTGCCGCTCAGACCGCTGCTGGTCGTCGTCAAGCTATGGAAATCATGAGGCAGACGAACATCCAGAATGCTGACCTATCGTTGCAAGCTCGAAGTAAACTTGAGGAAGCGTCCGCCGAGTTGACCCCACAGAACATGCAGAATGTCCaa                                                                                                    >  1:485575/1‑301 (MQ=255)`  
                                                                                                                                                                                                         `gCGGTATTCCATGAAGAGTACACACGAGATGGTCAATTTCAGTTATTTACAATCACGAAAGGAGGATAACCATATGTGTTGGGCAGCCGCAA‑‑‑TACCTATCGCTATATCTGGCGCTCAGGCTATCAGTGATCAGAACGCTCAGGCCAAAATGATTGCCGCTCAGACCGCTGCTGGTCGTCGTCAAGCTATGGAAATCATGAGGCAGACGAACATCCAGAATGCTGACCTATCGTTGCAAGCTCGAAGTAAACTTACGGAAGAGTCCGCCGAGTTGAACCCACCGAACATGCAGAAGTTCCaa                                                                                                    >  1:296799/1‑301 (MQ=255)`  
                                                                                                                                                                                                           `ggTATTCCATGAAGAGTACACACGAGATGGTCAATTTCAGTTATTTACAATCACGAAAGGAGGATAACCATATGTGTTGGGCAGCCGCAA‑‑‑TACCTATCGCTATATCTGGCGCTCAGGCTATCAGTGATCAGAACGCTCAGGCCAAAATGATTGCCGCTCAGACCGCTGCTGGTCGTCGTCAAGCTATGGAAATCATGAGGCAGACGAACATCCAGAATGCTGACCTATCGTTGCAAGCTCGAAGTAAACTTGAGGAAGCGTCCGCCGAGTTGACCCCACAGAACATGCAGAAGGTCCAAGc                                                                                                  <  1:507860/301‑1 (MQ=255)`  
                                                                                                                                                                                                            `gTATTCCATGAAGAGTACACACGAGATGGTCAATTTCAGTTATTGACAATCACGAAAGGAGGATAACCATATGTGTTGGGCAGCCGCAA‑‑‑TACCTATCGCTATATCTGGCGCTCAGGCTATCAGTGATCAGAACGCTCAGGCCAAAATGATTGCCGCTCAGACCGCTGCTGGTCGTCGTCAAGCTATGGAAATCATGAGGCAGACGAACATCCAGAATGCTGACCTATCGTTGCAAGCTCGAAGTAAACTTGAGGAAGCGTCCGCCGAGTTGACCCCACAGAACATGCAGAAGGTCCAAGCt                                                                                                 <  1:386177/301‑1 (MQ=255)`  
                                                                                                                                                                                                            `gTATTCCATGAAGAATACACACGAGATGGTCAATTTCAGTTATTTACAATCACGAAAGGAGGATAACCATATTTGTTGGGCAGCCGCAA‑‑‑TACCTATCGCTATATCTGGCGCTCAGGCTATCAGTGATCAGAACGCTCAGGCCAAAATGATTGCCGCTCAGACCGCTGCTGGTCGTCGTCAAGCTATTGAAATCATGAGGCAGACGAACATCCAGAATGCTGACCTAGCGTTGCAAGCTCGAAGTAAACTTGAGGAAGCGTCCGCCGAGTTGACCCCACAGAACATGCAGAAGGTCCAAGCt                                                                                                 <  1:167420/301‑1 (MQ=255)`  
                                                                                                                                                                                                             `tattcatggAAGAGTACACTCGAGAGTGTTAATTTCAGTGATTAACAATCACGAAAGGAGGATCACCATATGTGTTTGGCAGCCGCAA‑‑‑TACCGATCGCTAGATCGTGCGCTCAGGCTATCAGTGAGCAGAACGCTTAGGCCAAAGTGATTGCCGCTCAGACTGTTGCTGGTCGTCGTCAAGCTATGGAAATCATGAGGCAGACGAACATCCAGAATGCTGACCTAGCGTTGCAAGGTCGAAGTAAACTTGAGGAATAGTCCGCCGAGTGGACCTCACAGAACATGCAGAAGGTCCAAGCTa                                                                                                <  1:233238/293‑1 (MQ=255)`  
                                                                                                                                                                                                              `aTTCCATGAAGAGTACACACGAGATGGTCAATTTCAGTTATTTACAATCACGAAAGGAGGATAACCATATGTGTTGGGCAGCCGCAA‑‑‑TACCTATCGCTATATCTGGCGCTCAGGCTATCAGTGATCAGAACGCTCAGGCCAAAATTATTGCCGCTCAGACCGCTGCTGGTCGTCGTCACGCTATGGAAATCATGAGGCAGACGAACATCCAGAATGCTGACCTATCGTTGCAAGCTCGTAGTAAACTTGAGGAAGCGTCCGCCGAGTTGACCCCCCAGAACATGCAGAAggctgtatctta                                                                                               >  1:422761/1‑291 (MQ=255)`  
                                                                                                                                                                                                              `aTTCCATGAAGAGTACACACGAGATGGTCAATTTCAGTTATTTACAATCACGAAAGGAGGATAACCATATGTGTTGGGCAGCCGCAA‑‑‑TACCTATCGCTATATCTGGCGCTCAGGCTATCAGTGATCAGAACGCTCAGGCAAAAATGATTGCCGCTCAGACCGCTGCTGGTCGTCGTCAAGCTATGGAAATCATGAGGCAGACGAACATCCAGAATGCTGACCTATCGTTGCAAGCTCGAAGTAAACTTGAGGAAGCGTCCGCCGAGTTGACCTCACAGAACATGCAGAAGGTCCAAGCTAt                                                                                               >  1:302719/1‑301 (MQ=255)`  
                                                                                                                                                                                                                `tCCATGAAGAGTACACACGAGATGGTCAATTTCAGTTATTTACAATCCTGAAAGGAGGATAACCATATGTGTTGGGCAGCCGCAA‑‑‑TACCTATCGCTATATCTGGCGCTCAGGCTATCAGTGATCAGAACGCTCAGGCCAAAATGATTGACGCTCAGACCGCTGCTGGTCGTCGTCAAGCTATGGAAATAATGAGGCGGACGAACATCCAGAATGCTGACCTATCGTTGCAAGCTCGAAGTAAACTTGAGTAAGCGTCCGCCGAGTTGACCCCACAGAACCTgtctcttatacacatctccg                                                                                             >  1:124221/1‑282 (MQ=255)`  
                                                                                                                                                                                                                `tCCATGAAGAGTACACACGAGATGGTCAATTTCAGTTATTTACAATCACGAAAGGAGGATAACCATATGTGTTGGGCAGCCGCAA‑‑‑TACCTATCGCTATATCTGGCGCTCAGGCTATCAGTGATCAGAACGCTCAGGCCAAAATGCTTGCCGCTCAGACCGCTGCTGGTCGTCGTCAAGCTATGGAAATCCTGAGGCAGACGAACATCCAGAATGCTGACCTATCGTTGCAAGCTCGAAGTAAACTTGAGGAAGCGTCCGCCGAGTTGACCCCACAGAACATGCAGAAGGTCCAAGCTCTTg                                                                                             >  1:279227/1‑301 (MQ=255)`  
                                                                                                                                                                                                                `tCCATGAAGAGTACACACGAGATGGTCAATTTCAGTTATTTACAATCACGAAAGGAGGATAACCATATGTGTTGGGCAGCCGCAA‑‑‑TACCTATCGCTATATCTGGCGCTCAGGCTATCAGTGATCAGAACGCTCAGGCCAAAATGATTGCCGCTCAGACCGCTGCTGGTCGTCGTCAAGCTATGGAAATCATGAGGCAGACTAACATCCAGAATGCTGACCTATCGTTGCAAGCTCGAAGTAAACTTGAGGAAGCGTCCGCCGAGTTGACCCCACAGAACATGCAGAAGGTCCAAGCTATTg                                                                                             >  1:400714/1‑301 (MQ=255)`  
                                                                                                                                                                                                                  `cATGAAGAGTACACACGAGATGGTCAATTTCAGTTGTTTACAATAACGAAAGGAGGATAACCATATGTGTTGGGCAGCCGCAA‑‑‑TACCTATCGCTATATCTGGCGCTCAGGCTATCAGTGATCAGAACGCTCAGGCCAAAATGATTGCCGCTCAGACCGCTGCTGGTCGTCGTCAAGCTATGGAAATCATGAGGCAGACGAACATCCAGAATGCTGACCTATCGTTGCAAGCTCGAAGTAAACTTGAGGAAGCGTCCGCCGAGTTGACCTCACAGAACATGCAGAAGGTCCAAGCTATTggg                                                                                           <  1:2291/301‑1 (MQ=255)`  
                                                                                                                                                                                                                     `gAAGAGTACACACGAGATGGTCAATTTCAGTTATTTACAATCACGAAAGGAGGATAACCATATGTGTTGGGCAGCCGCAA‑‑‑TACCTATCGCTATATCTGGCGCTCAGGCTATCAGTGATCAGAACGCTCAGGCCAAAATGATTGCCGCTCAGACCGCTGCTGGTCGTCGTCAAGCTATGGAAATCATGAGGCAGACGAACATCCAGAATGCTGACCTATCGTTGCAAGCTCGAAGTAAACTTGAGGAAGCGTCCGCCGAGTTGACCCCACAGAACATGCAGAAGGTCCAAGCTATTGGGTcc                                                                                        >  1:63780/1‑300 (MQ=255)`  
                                                                                                                                                                                                                        `gagTACACACGAGATGGTCAATTTCAGTTATTTACAATCACGAAAGGAGGATAACCATATGTGTTGGGCAGCCGCAA‑‑‑TACCTATCGCTATATCTGGCGCTCAGGCTATCAGTGAACAGAACGCTCAGGCCAAAATGATTGCCGCTCAGAACGCTGCTGATCGTCGTAAAGCTATGGAAATAATGCGGCAGAAGAACATCCAGAATCCTGACCTATCGTTGCAAGCTCGAAGGAAACTTGAGGAAGCTTCTGCCGAGTTGACCCAAACAAACATGCAGAAGGTCCAAGCTATTTGGTCTATc                                                                                     >  1:68819/1‑301 (MQ=255)`  
                                                                                                                                                                                                                          `gTACACACGAGATGGTCAATTTCAGTTATTTACAATCACGAAAGGAGGATAACCATATGTGTTGGGCAGCCGCAA‑‑‑TACCTATCGCTATATCTGGCGCTCAGGCTATCAGTGATCAGAACGCTCAGGCCAAAATGATTGCCGCTCAGACCGCTGCTGGTCGTCGTCAAGCTATGGAAATCATGAGGCAGACGAACATCCAGAATGCTGAGCTATCGTTGCAAGCGGGAAGTAAACTTGAGGAAGGGTCAGCCGAGTGGAACTCACAGAACATGCAGAAGGTCAAAGCTATTGGGTCTATCCg                                                                                   >  1:367854/1‑301 (MQ=255)`  
                                                                                                                                                                                                                            `acacacGAGATGGTCAATTTCAGTTATTTACAATCACGAAAGGAGGATAACCATATGTGTTGGGCAGCCGCAA‑‑‑TACCTATCGCTATATCTGGCGCTCAGGCTATCAGTGCTCAGAACGCTCATGCCAAAATGATTGCCGCTCAGACCGCTGCTGGTCGTCGTCCAGCTATGGAAATCATGAGGCAGACGAACATCCAGAATGCTGACCTATCGTTGCAAGCTCGAAGTAAACTAGAGGAAGCGTCCGCCGAGTTGACCCCACAGAACATGCAGAAGGTCCAAGCTATTAGATCTATCCGAg                                                                                 >  1:76949/1‑301 (MQ=255)`  
                                                                                                                                                                                                                                          `aaaTGTCAGTTATTTACAATCACGAAAGGAGGATAACCATATGTGTTGGGCAGCCGCAA‑‑‑TACCTATCGCTATATCTGGCGCTCAGGCTATCAGTGATCAGAACGCTCAGGCCAAAATGATTGCCGCTCAGACCGCTGCTGGTCGTCGTCAAGCTATGGAAATCATGAGGCAGACGAACATCCAGAATGCTGACCTATCGTTGCAAGCTCGAAGTAAACTTGAGGAAGCGTCCGCCGAGTTGACCTCACAGAACATGCAGAAGGTCCAAGCTATTGGGTCTATCCGAGCGGCTATCGgagag                                                                   <  1:219414/300‑1 (MQ=255)`  
                                                                                                                                                                                                                                            `aTTTCAGTTATTTACAATCACGAAAGGAGGATAACCATATGTGTTGGGCAGCCGCAA‑‑‑TACCTATCGCTATATCTGGCGCTCAGGCTATCAGTGATCAGAACGCTCAGGCCAAAATGATTGCCGCTCAGACAGCTGCTGGTCGTCGTCAAGCTATGGAAATCATGAGGCAGACGAACATCCAGAATGCTGACCTATCGTTGCAAGCTCGAAGTAAACTTGAGGAAGCGTCCGCCGAGTTGACATCACAGAACATGCAGAAGGTCCAAGCTATTGGGTCTATCCGAGCGGCTATCGGagagag                                                                 >  1:312309/1‑301 (MQ=255)`  
                                                                                                                                                                                                                                             `tttCAGTTATTTACAATCACGAAAGGAGGATAACCATATGTGTTGGGCAGCCGCAA‑‑‑TACCTATCGCTATATCTGGCGCTCAGGCTATCAGTGATCAGAACGCTCAGGCAAAAATGATTGCCGCTCAGACCGCTGCTGGTCGTCGTCAAGCTATGGAAATCATGAGGCAGACGAACATCCAGAATGCTGACCTAGCGTTGCAAGCTCGAAGTAAACTTGAGGAAGCGTCTGCCGAGTTGACCTCACAGAACATGCAGAAGGTACAAGCTATTGGGTCTATCCGAGCGGCTATCGGAGAGAGt                                                                >  1:290603/1‑301 (MQ=255)`  
                                                                                                                                                                                                                                             `tttCAGTTATTTACAATCACGAAAGGAGGATAACCATATGTGTTGGGCAGCCGCAA‑‑‑TAACTATCGCTATATCTGGCGCTCAGGCTATCAGTGATCAGAACTCTCAGGCCAAAATGATTGCCGCTCAGACCGCTGCTGGTCGTCGTCAAGCTATGGAAATCATGAGGCAGACGAACATCCAGAATGCTGACCTATCGTTGCAAGCTCGAAGTCACCTTTAGGAAGCACCGGCCGTGTTGCCCCCACAGACCGTGCAGAAGGTCCAAGCTATTGGTTCTATCCGAGCGGCTGTCGtagaaatt                                                                >  1:108698/1‑297 (MQ=255)`  
                                                                                                                                                                                                                                             `tttCAGTTATTTACAATCACGAAAGGAGGATAACCATAGGTGTTGGGCAGCCGAAATACTACCGATCGCTATATCTGGCGCTCAGGCTATCAGTGATCAGAACGCTCAGGCCAAAATGATTGCCGCTCAGACCGCTGCTGGTCGTCGTCAAGCTATGGAAATCATGAGGCAGACGAACATCCAGAATGCTGACCTATCGTTGCAAGCTCGAAGTAAACTTGAGGAAGCGTCCGCCGAGTTGACCTCACAGAACATGCAGAAGGTCCAAGCTATTGGGTCTATCCGAGCGGCTATCGgagag                                                                   <  1:104973/301‑1 (MQ=255)`  
                                                                                                                                                                                                                                                       `ttACAATCACGAACGGAGGATAACCATATGTGTTGGGACGCCGCAA‑‑‑TACCGATCGCTATATCTGGCGCTCAGGCTATCAGTGATCAGAACGCTCACGCCAAAATGATTGCCGCTCCGACCGCTGCTGGTCGTCGTCAAGCTATGGCAATCATGAGGCAGACGAACATCCAGAATGCTGACCTATCGTTGCACGCTCGAAGTAAACTTGAGGAAGCGTCCGCCGAGTTGACCCCACAGAACATGCAGAAGGTCCAAGCTATTGGGTCTATCCGAGCGGCTATCGGAGAGAGTATGCTTGAAg                                                      <  1:39508/301‑1 (MQ=255)`  
                                                                                                                                                                                                                                                        `tACAAGCACGAAAGGAGGAGAACCATATGTGTTGGGCAGCCGCAA‑‑‑TACCTATCGCTATATCTGGCGCTCAGGCTATCAGTGATCAGAACGCTCAGGCCAAAATGATTGCCGCTCAGACCGCTGCTGGTCGTCGTCAAGCTATGGAAATCATGAGGCAGACGAACATCCAGAATGCTGACCTATCGTTGCAAGCTCGAAGTAAACTTGAGGAAGCGTCCGCCGAGTTGACCCCACAGAACATGCAGAAGGTCCAAGCTATTGGGTCTATCCGAGCGGCTATCGGAGAGAGTATGCTTGAAgg                                                     <  1:60867/301‑1 (MQ=255)`  
                                                                                                                                                                                                                                                            `aTCACGAAAGGAGGATAACCATATGTGTTGGGCAGCCGCAA‑‑‑TACCTATCGCTATATCTGGCGCTCAGGCTATCAGTGATCAGAACGCTCAGGCCAAAATGATTGCCGCTCAGACCGCTGCTGGTCGTCGTCAAGCTATGGAAATCATGAGGCAGACGAACATCCAGAATGCTGACCTATCGTTGCAAGCTCGAAGTAAACTTGAGGAAGCGTCCGCCGAGTTGACCCCACAGAACATGCAGAAGGTCCAAGCTATTGGGTCTATCCGAGCGGCTATCGgagagctgtctcttatacacatc                                                 >  1:356759/1‑283 (MQ=255)`  
                                                                                                                                                                                                                                                              `cACGAAAGGAGGATAACCATATGTGTTGGGCAGCCGCAA‑‑‑TACCTATCGCTATATCTGGCGCTCAGGCTATCAGTGATCAGAACGCTCAGGCTAAAATGATTGCCGCTCAGACCGCTGCTGGTCGTCGTCAAGCTATGGAAATCATGAGGCAGACGAACATCCAGAATGCTGACCTATCGTTGCAAGCTCGAAGTAAACTTGAGGAATCGTCCGCCGAGTCGACCCCACAGCACATGCAGAAGGCCCAAGCTATTGGGTCAATCCGAGCGGCTATCGGAGAGAGTATGCTTGAAGGttcttc                                               >  1:138900/1‑301 (MQ=255)`  
                                                                                                                                                                                                                                                              `cACGAAAGGAGGATAACCATATGTGTTGGGCAGCCGCAA‑‑‑TACCTATCGCTATATCTGGCGCTCAGGCTATCAGTGATCAGAACGCTCAGGCCAAAATGATTTCCGCTCAGACCGCTGCTGGTCGTCGTCAAGCTATGGAAATCATGAGGCAGACGAACATCCAGAATGCTGACCTATCGTTGCAAGCTCGAAGTAAACTTGAGGAAGCGTCCGCCGAGTTGACCCCACAGAACATGCAGAAGGTCCAAGCTATTTGGTCTATCCGAGCGGCTATCGGAGAGAGTATGCTTGACGGatcatc                                               >  1:72952/1‑301 (MQ=255)`  
                                                                                                                                                                                                                                                              `cACGAAAGGAGGATAACCATATGTGTTGGGCAGCCGCAA‑‑‑TACCTATCGCTATATCTGGCGCTCAGGCTATCAGTGATCAGAACGCTCAGGCCAAAATGATTGCCGATCAGACCGCTGCTGGTCGTCGTCAAGCTATGGAAATCATGAGGCAGACGAACATCCAGAATGCTGACCTATCGTTGCAAGCTCGAAGTAAACTTGAGGAAGCGTCCGCCGAGTTGACCTCACAGAACATGCAGAAGGTCCAAGCTATTGGGTCTATCCGAGCGGCTATCGGAGAGAGTATGcccgtcacttataa                                               >  1:16357/1‑288 (MQ=255)`  
                                                                                                                                                                                                                                                              `cACGAAAGGAGGATAACCATATGTGTTGGGCAGCCGCAA‑‑‑TACCTATCGCTATATCTGGAGCTCAGTCTATCAGTGATCAGAACGCTCAGTCCAAAATGATTGCCTCTCAGACCGCTGCTGGTCGTCGTCATGCTATGGAAATCATGTGGCAGACTAACATCCAGAATGCTGACCTATCGTTGCAATCTAGAAGTAAACTTGAGGAAGCGTCCGCCGAGTTGACCCCACAGAACATGCAGAAGGTCCAAGCTATTGGGTCTATCCGCGAGGCTATCGGAGAGAGTATGCTTGAAGTTTCCTc                                               >  1:286226/1‑301 (MQ=255)`  
                                                                                                                                                                                                                                                                     `ggaggaTAACCATATGTGTGGGGCAGGCGCAA‑‑‑TACCTATCGCTATATCTGGCGCTCAGGCTATCAGTGAACAGAACGATCAGGCCAAAATGATTGCCGCTCAGACCGCTGCTGGTAGTCGTCAAGCTATGGAAATCATGAGGCAGACGAACATCCAGAATGCTGACCTATCGTTGCAAGCTCGAAGTAAACTTGAGGAAGCGTCCGCCGAGTTGACCTCACAGAACATGCAGAAGGTCCAAGCTATTGGGTCTATCCGAGCGGCTATCTGAGAGAGTATGCTTGAAGGTTCCTCAATGGAc                                        <  1:114481/301‑1 (MQ=255)`  
                                                                                                                                                                                                                                                                      `gaggaTAACCATATGTGTTGGGCAGCCGCAA‑‑‑TACCTATCGCTATATCTGGCGCTCAGGCTATCAGTGATCAGAACGCTCAGGCCAAAATGATTGCCGCTCAGACCGCTGCTGGTCGTCGTCAAGCTATGGAAATCATGAGGCAGACGAACATCCAGAATGCTGACCTATCGTTGCAAGCTCGAAGTAAACTTGAGGAAGCGTCCGCAGAGTTGACCCCACAGAACATGCAGAAGGTCCAAGCTATTGGGTCTATCCGAGCGGCTATCGGAGAGAGTATGCTTGAAGGTTCCTCAATGGAcc                                       >  1:153535/1‑301 (MQ=255)`  
                                                                                                                                                                                                                                                                      `gaggaTAACCATATGTGTTGGGCAGCCGCAA‑‑‑TACCTATCGCTATATCTGGCGCTCAGGCTATCAGTGATCAGAACGCTCAGGACAAAATGATTGCCGCTCAGACCGCTGCTGGTCGTCGTCAAGCTATGGAAATCATGAGGCAGACGAACATCCAGAATGCTGACCTATCGTTGCAAGCTCGAAGTAAACTTGAGGAAGCGTCCGCCGAGTTGACCTCACAGAACATGCAGAAGGTCCAAGCTATTGGGTCTATCCGAGCGGCTATCGGAGAGAGTATGCTTGAAGGTTCCTCAATGGAcc                                       <  1:8702/301‑1 (MQ=255)`  
                                                                                                                                                                                                                                                                       `aggaTAACCATATGTGTTGGGCAGCCGCAA‑‑‑TACCTATCGCTATATCTGGCGCTCAGGCTATCAGTGATCAGAACGCTCAGGCCAAAATGATTGCCGCTCAGACCGCTGCTGGTCGTCGTCAAGCTATGGAAATCATGAGGCAGACGAACATCCAGCATGCTGACCTATCGTTGCAAGCTCGAAGTAAACTTGAGGAAGCGTCCGCCGAGTTGACCTCACAGAACATGCAGAAGGTCCAAGCTATTGGGTCTATCCGAGCGGCTATCGGAGCGATTCTGCTTGACGGTTCCTCAATGGACCg                                      >  1:495310/1‑301 (MQ=255)`  
                                                                                                                                                                                                                                                                        `ggaTAACCATATGTGTTGGGCAGCCGCAA‑‑‑TACCTATCGCTATATCTGGCGCTCAGGCTATCAGTGGTCAGAACGCTCAGGCCAAAATGATTGCCGCTCAGACCGCTGCTGGTCGTCGTCAAGCTATGGAAATCATGAGGCAGACGAACATCCAGAATGATGACCTATCGTTGCAAGCTCGAAGTAAACTTGAGGAAGCGTCCGCCGAGTTGACCCCACAGAACATGCAGAAGGTCCAAGCTATTGGGTCTATACGAGCGGCTATCGGAGAGAGTATGCTTGAAGGTTCCTCAATGGACCGc                                     >  1:192365/1‑301 (MQ=255)`  
                                                                                                                                                                                                                                                                        `ggaTAACCATATGTGTTGGGCAGCCGCAA‑‑‑TACCTATCGCTATATCTGGCGCTCAGGCTATCAGTGATCAGAACGCTCAGGCCAAAATGATTGCCGCTCAGACCGCTGCTGGTCGTCGTCAAGCTATGGAAATCATGAGGCAGACGAACATCCAGAATGCTGACCTATCGTTGCAAGCTCGAAGTAAACTTGAGGACGCGTCCGCCGAGTTGACCCCACAGAACATGCAGAAGGTCCAAGCTATTGGGTCTATCCGAGCGGCTATCGGAGAGAGTATGCTTGAAGGTTCCTCAATGGAccct                                     >  1:91890/1‑299 (MQ=255)`  
                                                                                                                                                                                                                                                                        `ggaTAACCATATGTGTTGGGCAGCCGCAA‑‑‑TACCTATCGCTATATCTGGCGCTCAGGCTATCAGTGATCAGAACGCTCAGGCCAAAATGATTGCCGCTCAGACCGCTGCTGGTCGTCGTCAAGCTATGGAAATCATGAGGCAGACGAACATCCAGAATGCTGACCTATCGTTGCAAGCTCGAAGTAAACTTGAGGAAGCGTCCGCCGAGTTGACCCCACAGAACATGCAGAAGGTCCAAGCTATTGGGTCTATCCGAGCGGCTATCGGAGAGAGGATGCTTGAAGGTTCCGCAATGGACCgg                                     >  1:158976/1‑300 (MQ=255)`  
                                                                                                                                                                                                                                                                        `ggaTAACCATATGTGTTAGGCAGCCGCAA‑‑‑TACCTATCGCTATATCTGGCGCTCAGGCTATCAGTGATCAGAACGCTCAGGCCAAAATGATTGCCGCTCAGACCGCTGCTGGTCGTCGTCAAGCTATGGAAATCATGAGGCAGACGAACATCCAGAATGCTGACCTATCGTTGCAAGCTCGAAGT‑AACTTGCGGAAGCGTCCGCCGAGTTGACCCCACAGAACATGCAGAAGGTCCAAGCTATTGGGTCTATCCGAGCGGCTATCGGAGAGAGTCTgtctcttatacacatctcctagcccc                                    >  1:183380/1‑276 (MQ=255)`  
                                                                                                                                                                                                                                                                           `tAACCATATGTGTTGGGCAGCCGCAA‑‑‑TACCTATCGCTATATCTGTCGCTCAGGCTATCAGTGATCAGAACGCTCAGGCCAAAATGATTGCCGCTCAGACCGCTGCTGGTCGTCGTCAAGCTATGGAAATCATGAGGCAGACGAACATCCAGAATGCTGACCTATCGTTGCAAGCTCGAAGTAAACTTGAGGAAGCGTCCGCCGAGTTGACCCCACAGAACATGCAGAAGGTCCAAGCTATTGGGTCTATCCGAGCGGCTATCGGAGAGAGTATGCTTGAAgctgtctcttatacacatctc                                  >  1:201572/1‑281 (MQ=255)`  
                                                                                                                                                                                                                                                                            `aaCCATATGTGTTGGGCAGCCGCAA‑‑‑TACCTATCGCTATATCTGGCGCTCAGGCTATCAGTGATCAGAACGCTCAGGCCAAAATGATTGCCGCTCAGACCGCTGCTGGTCGTCATCAAGCTATGGAAATCATGAGGCAGACGAACATCCAGAATGCTGACCTATCGTTGCAAGCTCGAAGTAAACTTGAGGAAGCGTCCGCCGAGTTGACCCCACATAACATGCATAAGGTCCAAGCTATTGGGTCTATCCGAGCGGCTATCGGAGAGAGTATGCTTTAAGGTTCCTCAATGGACCGCATTa                                 >  1:206791/1‑301 (MQ=255)`  
                                                                                                                                                                                                                                                                             `gccATATGTGTTGGGTAGCCGCAA‑‑‑GACCTCACGCTATATCTGGCGCTCAGGCTATCAGTTATCAGAACGCTCAGGCC‑AAATGATTGCCGCTCAGACCGCTGCTGGTCGTCGTCAAGCTATGGAAATCATGAGGCAGACGAACATCCAGCATGCTTACCTATCGTTGCAAGCTCGAAGTAAACTTGAGGAAGCGTCCGCCGAGTTGACACCTCCGAACATGCAGAAGGTCCAAGCTATTGGGTCTATCCGAGCGGCTATCGGAGAGAGTATGCTTGAAGGTTCCTCAATGGACCGCATTAAg                               <  1:81991/300‑1 (MQ=255)`  
                                                                                                                                                                                                                                                                             `aCCATATGTGTTGGGCAGCCGCAA‑‑‑TACCTATCGCTATATCTGGCGCTCAGGCTATCAGTGATCAGAACGCTCAGGCCAAAATGATTGCCGCCCAGACCGCTGCTGGTCGTCGTCAAGCTATGGAAATCATGAGGCAGACGAACATCCAGAATGCTGACCTATCGTTGCAAGCTCGAAGTAAACTTGAGGAAGCGTCCGCCGAGTTGACCTCACAGAACATGCAGAAGGTCCAAGCTATTGGGTCTATCCGAGCGGCTATCGGAGAGAGTATGCTTGAAGGTTCCTCAATGGACCGCATTaa                                >  1:347141/1‑301 (MQ=255)`  
                                                                                                                                                                                                                                                                              `ccATATGTGTTGGGCAGCCGCAA‑‑‑TACCTATCGCTATATCTGGCGCTCAGGCTATCAGTGATCAGAACGCTCATGCCAAAATGATTGCCGCTCAGACCGCTGCTGGTCGTCGTCAAGCTATGGAAATCATGAGGCAGACGAACATCCAGAATGCTGACCTATCGTTGCAAGCTCGAAGTAAACTTGAGGAAGCGTCCGCCGAGTTGACCCCACAGAACATGCAGAAGGTCCAAGCTATTGGGTCTATCCGAGCGGCTATCGGAGAGATTATGCTTGAAGGTTCCTCAATGGACCGCATTAAg                               >  1:283265/1‑301 (MQ=255)`  
                                                                                                                                                                                                                                                                              `ccATATGTGTTGGGCAGCCGCAA‑‑‑TACCTATCGCTATATCTGGCGCTCAGGCTATCAGTGATCAGAACGCTCAGGCCAAAATGATTGCCGCTCAGACCGCTGCTGGTCGTCGTCAAGCTATGGAAATCATGAGGCAGACGAACATCCAGAATGCTGACCTATCGTTGCAAGCTCGAAGTAAACTTGAGGAAGCGTCCGCCGAGTTGACCTCACAGAACATGCAGAAGGTCCAAGCTATTGGGTCTATCCGAGCGGCTATCGGAGAGAGTATGCTTGAAGGTTCCTCAATGGACCGCATTAAg                               <  1:329612/301‑1 (MQ=255)`  
                                                                                                                                                                                                                                                                              `ccATATGTGTTGGGCAGCCGCAA‑‑‑TACCTATCGCTATATCTGGCGCTCAGGCTATCAGTGATCAGAACGCTCAGGCCAAAATGATTGCCGCTCAGACCGCTGCTGGTCGTAGTCAAGCTATGGAAATCATGAGGCAGACGAACATCCAGAATGCTGACCTATCGTTGCAAGCTCGAAGTAAACTTGAGGAAGCGTCCGCCGAGTTGACCCCACAGAACATGCAGAAGGTCCAAGCTATTGGGTCTATCCGAGCGGCTATCGGAGAGAGTAGGCTTGAAGGTTCCTCAATGGACCGCATTAAg                               >  1:466710/1‑301 (MQ=255)`  
                                                                                                                                                                                                                                                                              `ccATATGTGTTGGGCAGCCGAAA‑‑‑TACCTATCGCTATATCTGGCGCTCAGGCTACCAGTGATCAGAACGATCAGGCCAAAATGATTGCAGCTCAGACCGCTGCTGGTCGTCGTCAAGCTATGGAAATCATGAGGCCGACGACCATCCAGAATGCTCACCTATCGTTGCAAGCTCGAAGTACACTTGAGGAAGCGGCCGCCGAGTTGACCTCACAGCACATGCAGAAGGTACAAGCTATTGGGTCTATCCGAGCAGCTATCGGAGAGAGTCTGCCTGCAGGTTACTCAATGGCCCACATTAAg                               >  1:96181/1‑301 (MQ=255)`  
                                                                                                                                                                                                                                                                               `cATATGTGTTGGGCAGCCGCAA‑‑‑TACCTATCGCTATATCTGGCGCTCAGGCTATCAGTGATCAGAACGCTCAGGCCAAAATGATTGCCGCTCAGACCGCTGCTGGTCGTCGTCAAGCTATGGAAATCATGAGGCAGACGAACATCCAGAATGCTGACCTATCGTTGCAAGCTCGAAGTAAACTTGAGGAAGCGTCCGCCGAGTTGACCCCACAGAACATGCAGAAGGTCCAAGCTATTGGGTCTATCCGAGCGGCTATCGGAGAGAGTATGCTTGAAGGTTCCTCAATGGACCGAATTAAGc                              <  1:171375/301‑1 (MQ=255)`  
                                                                                                                                                                                                                                                                               `cATATGGGTTGGGCAGCCGCAA‑‑‑TACCTATCGCTATATCTGGCGCTCAGGCTATCAGTGATCAGAACGCTCAGGCCAAAATGATGGCCGCTCAGACCGCTGCTGGTCGTCGTCAAGCTATGGAAATCATGAGGCAGACGAACATCCAGAATGCTGACCTATCGTTGCAAGCTCGAAGTAAACTTGAGGAAGCGTCCGCCGAGTTGACCCCACAGAACATGCAGAAGGTCCAAGCTATTGGGTCTATCCGAGCGGCTATCGGAGAGAGTATGCTTGAAGGTTCCTCAATGGACCGCATTAAGc                              <  1:264536/301‑1 (MQ=255)`  
                                                                                                                                                                                                                                                                                 `tatGTGTTGGGCAGCCGCAA‑‑‑TACCTATCGCTATATCTGGCGCTCAGGCTATCAGTGATCAGAACGCTCAGGCCAAAATGATTGCCGCTCAGACCGCTGCTGGTCGTCGTCAAGCTATGGAAATCATGAGGCAGACGAACATCCAGAATGCTGACCTATCGTTGCAAGCTCGAAGTAAACTTGAGGAAGCGTCCGCCGAGTTGACACCACAGAACATGCAGAAGGTCCAAGCTATTGGGTCTATCCGAGCGGCTATCGGAGAGAGTATGCTTGAAGGTTCCTCAATGGACCGCATTAAgagc                            >  1:249603/1‑298 (MQ=255)`  
                                                                                                                                                                                                                                                                                    `gtgtTGGGCAGCCGCAA‑‑‑TACCTATCGCTATATCTGGCGCTCAGGCTATCAGTGATCAGAACGCTCAGGCCAAAATGATTGCCGCTCAGACCGCTGCTGGTCGTCGTCAAGCTATGGAAATCATGAGGCAGACGAACATCCAGAATGCTGACCTATCGTTGCAAGCTCGAAGTAAACTTGAGGAAGCGTCCGCCGAGTTGACCTCACAGAACATGCAGAAGGCCCAAGCTATTTGGTCTATCCGAACGGCTATCGGAGAGAGTATGCTTGAAGGTTCCTCAATGGACCGCATTAAGCGAGTc                         >  1:48064/1‑301 (MQ=255)`  
                                                                                                                                                                                                                                                                                    `gtgtTGGGCAGCCGCAA‑‑‑TACCTATCGCTATATCTGGCGCTCAGGCTATCAGTGATCAGAACGCTCAGGCCAAAATGATTGCCGCTCAGACCGCTGCTGGTCGTCGTCAAGCTATGGAAATCATGAGGCAGACGAACATCCAGAATGCTGACCTATCGTTGCAAGCTCGAAGTAAACTTGAGGAAGCGTCCGCCGAGTTGACCCCACAGAACATGCAGAAGGTCCAAGCTATTGGGTCTATCCGAGCGGCTATCGGAGAGAGTATGCTTGAAGGTTCCtgtctcttatacacatctccgagc                         >  1:180427/1‑278 (MQ=255)`  
                                                                                                                                                                                                                                                                                    `gtgtTGGGCAGCCGCAA‑‑‑TACCTATCGCTATATCTGGCGCTCAGGCTATCAGTGATCAGAACGCTCAGGCCAAAATGATTGCCGCTCAGACCGCTGCTGGTCGTCGTCAAGCTATGGAAATCATGAGGCAGACGAACATCCAGAATGCTGACCTATCGTTGCAAGCTCGAAGTAAACTTGAGGAAGCGTCCGCCGAGTTGACCCCACAGAACATGCAGAAGGTCCAAGCTATTGGGTCTATCCGAGCGGCTATCGGAGAGAGTATGCTTGAAGGTTCCTCAATGGACCGCATTAAGCGAGTc                         <  1:415890/301‑1 (MQ=255)`  
                                                                                                                                                                                                                                                                                         `ataagagacaga‑‑‑TACCTATCGCTATATCTGGCGCTCAGGCTATCAGTGATCAGAACGCTCAGGCCAAAATGATTGCCGCTCAGACCGCTGCTGGTCGTCGTCAAGCTATGGAAATCATGAGGCAGACGAACATCCAGAATGCTGACCTATCGTTGCAAGCTCGAAGTAAACTTGAGGAAGCGTCCGCCGAGTTGACCTCACAGAACATGCAGAAGGTCCAAGCTATTGGGTCTATCCGAGCGGCTATCGGAGAGAGTATGCTTGAAGGTTCCTAAATGGACCGCATTAAGCGAGTCACAGa                    <  1:236674/290‑1 (MQ=255)`  
                                                                                                                                                                                                                                                                                              `gCCGCAA‑‑‑TACCTATCGCTATATCTGGCGCTCAGGCTATCAGTGATCAGAACGCTCAGGCCAAAATGATTGCCGCTCAGACCGCTGCTGGTCGTCGTCAAGCTATGGAAATCATGAGGCAGACGAACATCCAGAATGCTGACCTATCGTTGCAAGCTCGAAGTAAACTTGAGGAAGCGTCCGCCGAGTTGACCCCACAGAACATGCAGAAGGTCCAAGCTATTGGGTCTATCCGAGCGGCTATCGGAGAGAGTATGCTTGAAGGTTCCTCAATGGACCGCCTTTAGCGAGTCCCTGAAGGAc               >  1:491086/1‑301 (MQ=255)`  
                                                                                                                                                                                                                                                                                                `ggCAA‑‑‑GACCTATCGCGATATCTGGCGCTCAGGCTATCAGTGATCGGAACGCTCAGGCCAAAAGGATCGCCGCACAGACCGCTGATAGTCGACGTCAAGCGATGGAAATCATGAGGCAGACGAAGATGCAGAATGCTGACCGAACGTTGCAAGCTCGAAGTAAACTTGAGGAAGCGTCCGCCGAGTGGACCCAACAGAACAGGCAGAAGGTACAAGCGATTGGGTCTATCCGAGCGGCTATCGGAGAGAGTATGCTTGAAGGTTCCTCAATGGACCGCATTAAGCGAGTCACAGAAGGACAg             <  1:462435/300‑1 (MQ=255)`  
                                                                                                                                                                                                                                                                                                 `ttataaaggacagtCGCTATGACTGTCGCGCAGGCAATCAGTTATCAGAACGCTGAGGCCAATATGATTGCCGCGCAGACCGCTGCTGTTCGTCGTCAAGCGATGGAAATCATGAGGAAGACGAACATCCAGAATGTTGACCTATCGTTGCAAGCTCGAAGTAAACTTGAGGAAGCGTCCTCCGATTTGACCCCACAGAACATTCAAAAGGTCCAAGCTATTGGGTCTATCCAAGCGGCTATCGTAGAGAGTATGCTTGAAGGTTCCTCAATGGACCGCATTAAGCGAGTCACAGAAGGAc               <  1:50066/288‑1 (MQ=255)`  
                                                                                                                                                                                                                                                                                                         `aCCTATCGCTATATCTGGCGCTCAGGCTATCTGTGATCCGAACGCTCAGGCCTAAATGATTGCCGCTCAGACCGCTTCTGGTCGTCGTCAAGCTATGGAAATCATGAGGCAGACGAACATCCAGAATGCTGACCTATCGTTGCAAGCTCGAAGTAAACTTGAGGAAGCGTCCGCCGAGTTGACCCCACAGAACATGCAGAAGGTCCAAGCTATTGGGTCTATCCGAGCGGCTATCGGAGAGAGTATGCTTGAAGGTTCCTCAATGGACCGCATTAAGCGAGTCACAGAAGGACAGTTCAtt       <  1:244082/301‑1 (MQ=255)`  
                                                                                                                                                                                                                                                                                                         `aCCTATCGCTATATCTGGCGCTCAGGCTATCAGTGATCAGAACGCTCAGGCCAAAATGATTGCCGCTCAGACCGCTGCTGGTCGTCGTCAAGCTATGGAAATCATGAGGCAGACGAACATCCAGAATGCTGACCTATCGTTGCAAGCTCGAAGTAAACTTGAGGAAGCGTCCGCCGAGTTGACCTCACAGAACATGCAGAAGGTCCAAGCTATTGGGTCTATCCGAGCGGCTATCGGAGAGAGTATGCTTGAAGGTTCCTCAATGGACCGCATTAAGCGAGTCACAGAAGGACAGTTCAtt       >  1:341871/1‑301 (MQ=255)`  
                                                                                                                                                                                                                                                                                                         `aCCTATCGCTATATCTGGCGCTCAGGCTATCAGTGATCAGAACGCTCAGGCCAAAATGATTGCCGCTCAGACCGCTGCTGGTCGTCGTCAAGCTATGGAAATCATGAGGCAGACGAACATCCAGAATGCTGACCTATCGTTGCAAGCTCGAAGTAAACTTGAGGAAGCGTCCGCCGAGTTGACCCCACAGAACATGCAGAAGGTCCAAGCTATTGGGTCTATCCGAGCGGCTATCGGAGAGAGTATGCTTGAAGGTTCCTCAATGGACCGCATTAAGCGAGTCACAGAAGGACAGTTctgt       >  1:259591/1‑298 (MQ=255)`  
                                                                                                                                                                                                                                                                                                             `aTCGCTATATCTGGCGCTCAGGCTATCAGTGATCAGAACGCTCAGGCCAAAATGATTGCCGCTCAGACCGCTGCTGGTCGTCGTCAAGCTATGGAAATCATGAGGCAGACGAACATCCAGAATGCTGACCTATCGTTGCAAGCTCGAAGTAAACTTGAGGAAGCGTCCGCCGAGTTGACCCCACAGAACATGCAGAAGGTCCAAGCTATTGGGTCTATCCGAGCGGCTATCGGAGAGAGTATGCTTGAAGGTTCCTCAATGGACCGCATTAAGCGAGTCACAGAAGGACAGTTCATTCggg   <  1:134746/301‑1 (MQ=255)`  
                                                                                                                                                                                                                                                                                                             `aTCGCTATATCTGGCGCTCAGGCTATCAGTGATCAGAACGCTCAGGCCAAAATGATTGCCGCTCAGACCGCTGCTGGTCGTCGTCAAGCTATGGAAATCATGAGGCAGACGAACATCCAGAATGCTGACCTATCGTTGCAAGCTCGAAGTAAACTTGAGGAAGCGTCCGCCGAGTTGACCCCACAGAACATGCAGAAGGTCCAAGCTATTGGGTCTATCCGAGCGGCTATAGGAGAGAGTATGCTTGAAGGTTCCTCAATGGACCGCATTACGCGAGTCACAGAAGGACAGTTCATTCggg   >  1:222904/1‑301 (MQ=255)`  

                                                                                                                                                                                                                                                                                                              `|`                                                                                                                                                                                                                                                                                                              
`CTAGCTATCGGTGGTAACTGCGGGGACCAGTGCTGGTTCGTTACGAGCGACCAAGTGTGGCGACTTAGTGGAAAGGCTAAGCGAAAGTTCCGTAAGTTAATCATGGAGTATCGCGATAAGATGCTTGAGAAGTATGATACTCTTTGGAATTACGTATGGGTAGGCAATACGTCCCACATTCGTTTCCTCAAGACTATCGGTGCGGTATTCCATGAAGAGTACACACGAGATGGTCAATTTCAGTTATTTACAATCACGAAAGGAGGATAACCATATGTGTTGGGCAGCCGCAA‑‑‑TACCTATCGCTATATCTGGCGCTCAGGCTATCAGTGGTCAGAACGCTCAGGCCAAAATGATTGCCGCTCAGACCGCTGCTGGTCGTCGTCAAGCTATGGAAATCATGAGGCAGACGAACATCCAGAATGCTGACCTATCGTTGCAAGCTCGAAGTAAACTTGAGGAAGCGTCCGCCGAGTTGACCTCACAGAACATGCAGAAGGTCCAAGCTATTGGGTCTATCCGAGCGGCTATCGGAGAGAGTATGCTTGAAGGTTCCTCAATGGACCGCATTAAGCGAGTCACAGAAGGACAGTTCATTCGGGA  >  NC_001604.1/27454‑28053`

  

Alignment Legend

Aligned base mismatch/match (shaded by quality score): `ATCG/ATCG` `< 3 ≤` `ATCG/ATCG` `< 9 ≤` `ATCG/ATCG` `< 20 ≤` `ATCG/ATCG` `< 33 ≤` `ATCG/ATCG` `< 38 ≤` `ATCG/ATCG`

Unaligned base: `atcg`    Masked matching base: `atcg`    Alignment gap: `‑`    Deleted base: `‑`

Reads not counted as support for junction

read\_name Not counted due to insufficient overlap past the breakpoint.

read\_name Not counted due to not crossing MOB target site duplication.
